# Plicidentine in the Early Permian Parareptile *Colobomycter pholeter*, and Its Phylogenetic and Functional Significance among Coeval Members of the Clade

**DOI:** 10.1371/journal.pone.0096559

**Published:** 2014-05-07

**Authors:** Mark J. MacDougall, Aaron R. H. LeBlanc, Robert R. Reisz

**Affiliations:** Department of Biology, University of Toronto Mississauga, Mississauga, Ontario, Canada; Raymond M. Alf Museum of Paleontology, United States of America

## Abstract

Once thought to be an exclusively anamniote characteristic, plicidentine, a pattern of infolding of dentine, is now known to be found in various amniote clades, including Parareptilia. In the absence of detailed analyses of parareptilian dentition, most parareptiles were assumed to lack plicidentine due to the absence of external indicators, such as plications on the tooth base. The clear presence of this dentinal feature in the largest premaxillary and maxillary teeth of *Colobomycter pholeter*, led us to the present detailed study within the dentition of this unusual parareptile, and those of coeval members of this clade. Our study reveals that there is large variability in the degree of dentine infolding within *C. pholeter* dentition, as well as within those of closely related parareptiles. This variability ranges from a lack of plications, to very complex anamniote-like plicidentine. Utilizing computed tomography scans in conjunction with histological sections we also demonstrate the utility of computed tomography scans in conducting non-destructive sampling in the identification of plicidentine. Given the variability of plicidentine in this sample of parareptiles, we hypothesize that one function of parareptilian plicidentine is to increase the surface area for attachment tissues, and we suggest that the use of plicidentine as a character in phylogenetic analyses of parareptiles may be misleading.

## Introduction

Parareptilia, the sister taxon to Eureptilia, was a clade of amniotes that lived during the Palaeozoic and Early Mesozoic Eras. Parareptiles were uncommon members of most Early Permian communities, but by the Middle and Late Permian, parareptiles had obtained a global distribution, becoming integral components of terrestrial tetrapod communities [1], [2]. Although most were fairly small-bodied, parareptiles were strikingly diverse, ranging in form from small, superficially lizard-like predators to large armored herbivores. However, the Permo-Triassic extinction event resulted in a significant decline in the number of parareptilian lineages, with only one clade, the Procolophonoidea, surviving into the Mesozoic and eventually going extinct by the end of the Triassic [3]–[5].

Despite morphological differences between the major groups of parareptiles, the exact phylogenetic positions of some remain contentious [6]–[11]. The discrepancy in interpretations of their interrelationships illustrates the need for more phylogenetically informative characters. Recent studies have focused on parareptilian dentitions and tooth implantation [12], [13] and have highlighted the potential for differences in dental anatomy and attachment within several parareptilian groups to be informative in this regard. One of these features is the presence of plicidentine [14]. Plicidentine is a structural term that refers to the infolding of the dentine around the pulp cavity [15], [16], and its presence has historically been attributed to several anamniote groups, including labyrinthodont amphibians [17]. Plicidentine was first described by Owen [18] in the temnospondyl *Mastodonsaurus*, and was subsequently described in other anamniotes. The broad distribution of plicidentine (commonly referred to as “labyrinthine infoldings” sensu [18]) in amphibians, coupled with the absence of plicidentine in most derived groups of amniotes, has led many authors to suggest that plicidentine was lost early in the evolutionary history of Amniota [19], [20]. However, over the last century plicidentine has been documented in ichthyosaurs [21], choristoderes [22], mosasaurs [23], varanoids [24], snakes [25], captorhinids [26], and of particular interest, in a single parareptile, *Colobomycter pholeter* [14].

*Colobomycter pholeter* is an Early Permian (289 ma) parareptile from the Richards Spur locality in Oklahoma, USA. This locality is unique in preserving multiple taxa of small parareptiles, in strong contrast to all other Early Permian localities, where parareptiles are either absent, or represented by the remains of single taxa. The Dolese Brothers Limestone Quarry near Richards Spur has yielded the remains of several cranial fragments of *C. pholeter*, a second, undescribed species of this genus (personal observation, RRR), at least two species of *Delorhynchus* [27], [28], the enigmatic *Feeserpeton oklahomensis*, as well as *Microleter mckinzieorum*, all basal parareptiles. In addition, the more derived parareptile taxon *Bolosaurus grandis*, is known from jaw fragments. This unprecedented diversity of coeval parareptiles provides the basis of the current study, allowing us to compare in great detail the anatomy of the teeth of relatively closely related taxa.

Among early parareptiles, *C. pholeter* is readily distinguishable by the presence of two greatly enlarged maxillary and a single, enormous premaxillary tooth [14], [29]. In the case of the enlarged premaxillary tooth, it is much larger than any of the other teeth in the jaws, significantly surpassing even the size of the enlarged maxillary teeth. The unique dentition of *C. pholeter* is unlike any other found within the Palaeozoic, or even among reptiles. As previously mentioned, Modesto and Reisz [14] were able to determine the presence of plicidentine within the maxillary teeth of *C. pholeter*. This determination was possible because externally the bases of the teeth possessed the well developed plications characteristic of plicidentine. More importantly, many of the teeth were broken, revealing the presence of folded dentine. However, other than determining that plicidentine was present in *C. pholeter* they were able to say little else regarding the nature of the plicidentine, as histological analysis is required to determine the finer details of tooth tissues. The presence of plicidentine within *C. pholeter* combined with its unique and poorly studied dentition warranted a much more thorough examination of its teeth through histological examination.

Currently, histological examinations of parareptilian teeth have only been conducted on the procolophonid *Soturnia caliodon* [12], the mesosaur *Stereosternum tumidum* [30], the procolophonid *Procolophon trigoniceps* [31], and an indeterminate pareiasaur [31]. However, none of these studies was able to determine the nature and extent of the plicidentine in these animals. This is largely a result of the plane of section of the teeth that underwent histological analysis for the two former taxa, and an emphasis on studying enamel microstructure in the two latter taxa. The documentation of tooth tissues in longitudinal section makes the identification of plicidentine difficult, because the folds generally run parallel to the long axis of the tooth. In order for the infolding of the dentine (or lack thereof) to be clearly visible, and to document its interactions with the adjacent attachment tissues, it is necessary to take serial cross-sections of teeth as well as longitudinal sections through the tooth bearing elements.

**Figure 1:**
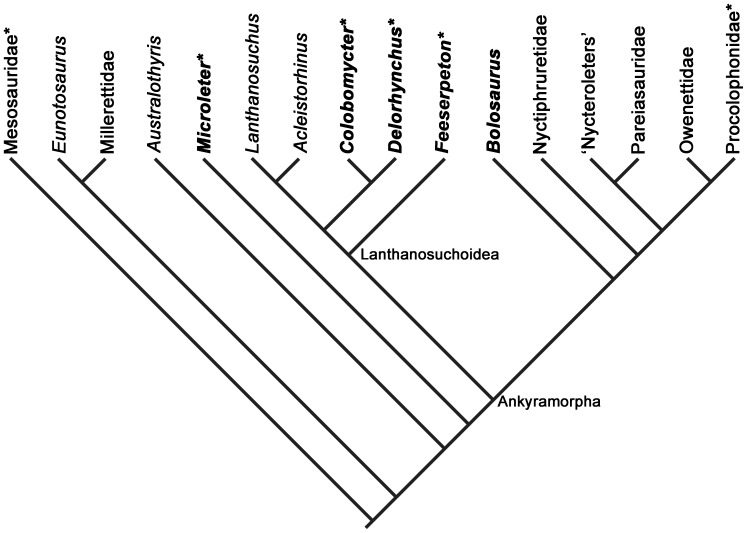
Cladogram showing the relationships of sampled parareptile taxa. Bolded taxa represent those sampled in this study. Taxa marked with an asterisk represent taxa that are known to exhibit plicidentine. The cladogram was modified from unpublished data of a forthcoming analysis, that itself was a modification of the MacDougall and Reisz [2] and Reisz et al. [27] analyses.

Here we present the first comparative histological examination of plicidentine in Parareptilia through the examination of *Colobomycter pholeter* and several of its contemporaneous relatives (Figure 1). We also demonstrate the potential use of X-ray micro-computed tomography (CT) scans for the study of different types of plicidentine, and hence avoidance of destructive methods. We also provide the first survey of the distribution of plicidentine in coeval parareptilian taxa, including Bolosauridae, three members of Lanthanosuchoidea, and *Microleter mckinzieorum*, and assess the phylogenetic and functional significance of parareptilian plicidentine.

## Materials and Methods

The data presented here was obtained from the study of five different parareptile taxa: *Colobomycter pholeter* Vaughn [29] (e.g., [32]), *Bolosaurus striatus* Reisz et al. [33], *Microleter mckinzieorum* Tsuji et al. [1], *Feeserpeton oklahomensis* MacDougall and Reisz [2], and a new species of *Delorhynchus* Reisz et al. [27]. With the exception of *B. striatus*, these taxa are all found at the Richards Spur locality of Oklahoma, USA, and in the case of *B. striatus* its dentition is similar in shape and age to that of *Bolosaurus grandis*, making these comparisons valid. Of these taxa, only *C. pholeter*, *B. striatus*, *M. mckinzieorum*, and the new species of *Delorhynchus* were chosen for histological preparation. *F. oklahomensis* (which is currently known from only a single specimen) was studied using CT scans and the imaging software Avizo 7.

Permission was obtained from all of the applicable institutions (Sam Noble Museum of Natural History in Norman, Oklahoma; University of Washington Burke Museum in Seattle, Washington; Goldfuβ Museum, Bonn, Germany; Royal Ontario Museum in Toronto, Canada) to borrow and work on the specimens that are presented herein. All specimens were loaned to R. R. Reisz with permission for preparation, and in the case of those that were thin-sectioned, histological analysis.

Material that was thin-sectioned included two dentary fragments of *Bolosaurus striatus* from the Lower Permian of Texas (StlPB-R 636, StlPB-R 637), as well as a partial maxilla (ROM 67372) and dentary (ROM 67373) of *Delorhynchus* cf. *Delorhynchus priscus*, a partial maxilla (ROM 67375) of *Microleter mckinzieorum*, and a premaxilla (UWBM 95405) and partial maxilla (ROM 67374) of *Colobomycter pholeter* from the Lower Permian of Oklahoma. Specimens were embedded in Castolite AP polyester resin under vacuum, and then left to dry for a 24-hour period. The specimens were then cut using a Buehler Isomet 1000 wafer blade low-speed saw. Cut specimens were then mounted to glass or plexiglass slides using Scotch-Weld SF-100 cyanoacrylate. The mounted specimens were ground down to approximately 180 µm thick using a Hillquist grinding cup, and then further ground manually using progressively finer grits of silicon carbide powder; lastly specimens were polished using one-micron grit aluminum oxide powder. Photography of specimens was performed using a Nikon DS-Fi2 camera mounted to a Nikon AZ-100 microscope fitted with crossed-polarizing filters, as well as an oblique illumination slider. Image processing was performed using Nikon NIS-Elements (Basic Research) imaging software registered to R. R. Reisz of the University of Toronto Mississauga.

CT scanning of *Colobomycter pholeter* was performed at the University of Calgary, and the CT scanning of *Feeserpeton oklahomensis* was performed at the University of Texas High Resolution X-ray CT Facility. The CT data set for *C. pholeter* consists of 950 slices at a resolution of 1120×1120 pixels, and the data set for *F. oklahomensis* consists of 623 slices at a resolution of 1024×1024 pixels. The slices were then imported into and manipulated using the imaging software Avizo 7 registered to R. R. Reisz of the University of Toronto Mississauga.

## Results

### *Colobomycter pholeter*

**Figure 2:**
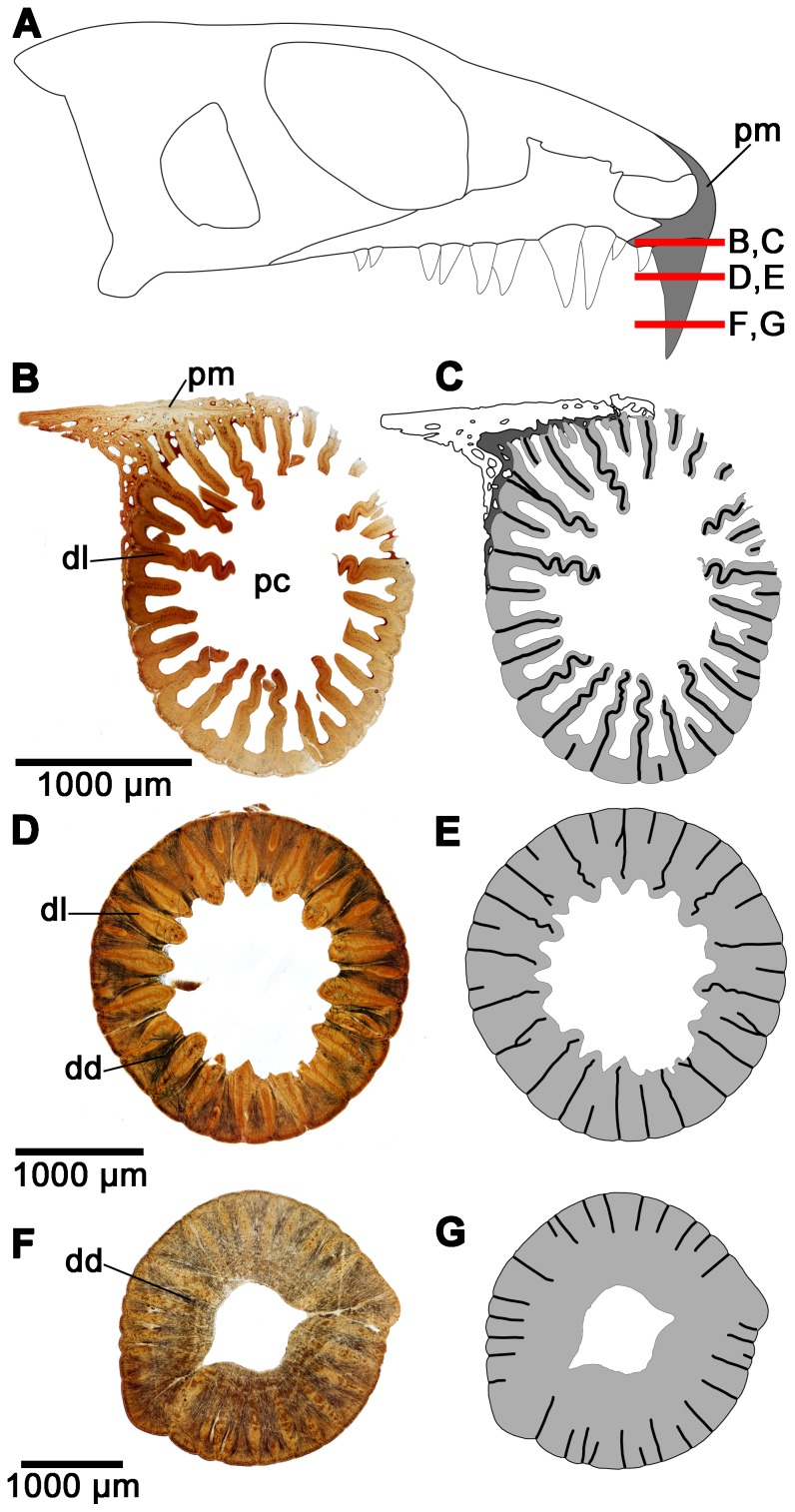
Cross-sectional views of the enlarged premaxillary tooth of *Colobomycter pholeter* (UWBM 95405). A: diagram indicating from what parts of the premaxilla the histological sections were taken. B: cross-section taken towards the base of the enlarged premaxillary tooth showing the complex infolding of the dentine and the presence of lamellae. C: interpretation of the cross-section in B. Light grey areas indicate dentine, while darker grey areas indicate alveolar bone. The thick black lines represent the axes of the folds. D: cross-section taken midway up the enlarged premaxillary tooth showing the presence of dark dentine. E: interpretation of the cross-section in D. Light grey areas indicate dentine, while the thick black lines represent the axes of the folds. F: cross-section taken towards the tip of the enlarged maxillary tooth. G: interpretation of the cross-section of F. Light grey areas indicate dentine, while the thick black lines represent the axes of the folds. dd, dark dentine; dl, dentine lamellae pc, pulp cavity; pm, premaxilla.

**Figure 3:**
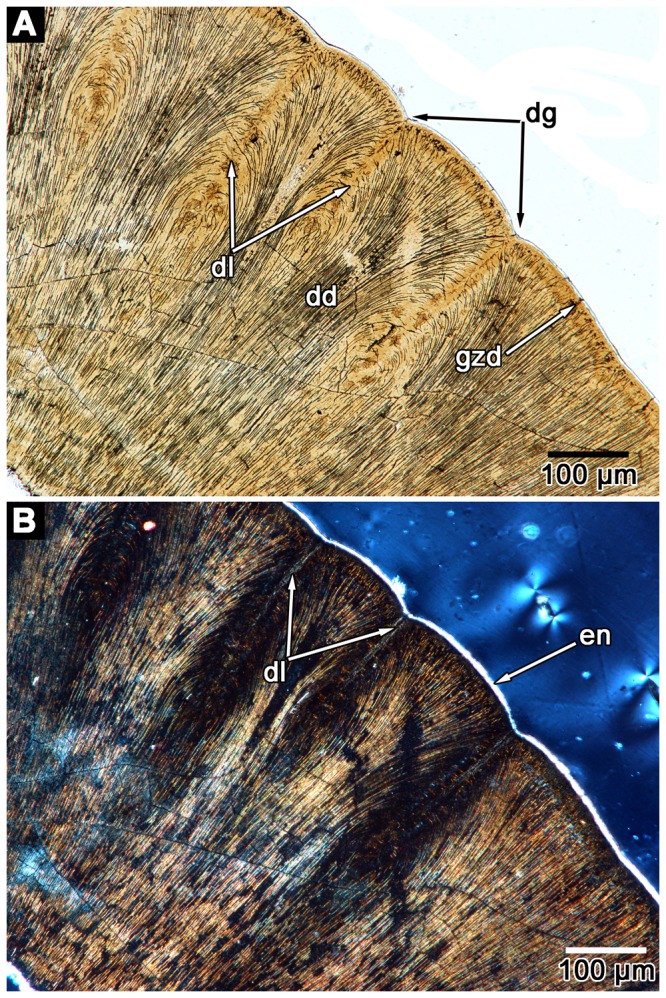
Microstructure in the crown of the enlarged premaxillary tooth of *Colobomycter pholeter* (UWBM 95405). A: Crown of the enlarged premaxillary tooth under polarized light. Note the presence of the globular zone of dentine, which tightly infolds to form lamellae. Also visible are dark dentine and the slight grooves formed in the dentine by the folding. B: same image as in A, but under cross-polarized light. Note the presence the thin enamel layer on the exterior of the tooth. dd, dark dentine; dg, dentine groove; dl, dentine lamellae; en, enamel; gzd, globular zone of the dentine.

As previously indicated, *Colobomycter pholeter* is unique among parareptiles, and Paleozoic reptiles in general, in that it possesses a pair of greatly enlarged maxillary teeth and a single, very large tooth on the premaxilla (Figure 2A). The remaining teeth of the maxilla and the single other premaxillary tooth are small and homodont. Modesto and Reisz [14] described plications on the exterior of the enlarged premaxillary and maxillary teeth, and broken maxillary teeth revealed that the walls of the tooth bases were folded. The thin sections of the teeth of *C. pholeter* reveal the presence of extensive plicidentine in cross-section. In particular, the dentine at the base of the enlarged premaxillary tooth of *C. pholeter* is arranged into a series of tight folds that radiate towards the pulp cavity (Figure 2B, C). The central component of each fold is composed solely of dentine, signifying that these dentine folds, termed lamellae [24], are so highly infolded that no attachment tissues from the outside of the tooth base contribute to the fold. The presence of convoluted folding and lamellae is very reminiscent of the complex plicidentine seen in labyrinthodont amphibians [34]. In *C. pholeter*, short, unbranched folds alternate with folds that are considerably longer and sinuous (Figure 2B–E). The midsection of the enlarged premaxillary tooth reveals that the folds maintain their complexity well above the jaw line. They exhibit the same alternating pattern of short and long lamellae seen lower in the tooth, although the spaces between lamellae are now occupied by dentine. Several of the lamellae exhibit primary branching, something that is not observed at the base of the tooth. Dentine tubules extending from the primary folds converge and form dark dentine. A byproduct of the dark dentine is the formation of lighter petaloid dentine, similar to that observed in embolomeres and temnospondyls [17]. Thin sections near the tip of the crown still exhibit plicidentine (Figure 2F, G). The folds are simpler, consisting of very short lamellae and none of the complex folding seen in lower sections through the tooth. The alternation between shorter and longer folds is largely maintained. Dark dentine is still apparent, although it is not quite as concentrated as it is in the midsection of the tooth. Due to the presence of lamellae in the crown of the tooth, the enamel that surrounds the tooth crown never enters into any of the folds of the tooth. However, the folding results in the enamel being grooved slightly wherever the folds are occurring (Figure 3).

**Figure 4:**
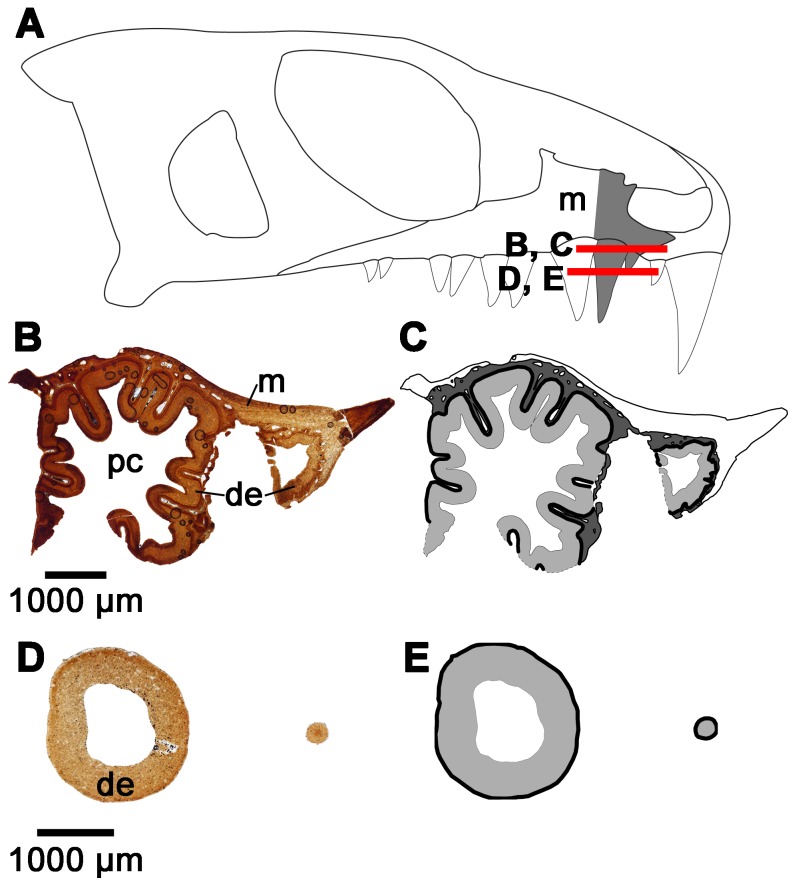
Cross-sectional views of the maxillary teeth of *Colobomycter pholeter* (ROM 67374). A: diagram indicating from what parts of the maxilla the histological sections were taken. B: cross-section taken towards the base of the maxillary teeth showing the convoluted dentine infolding of one of the enlarged maxillary teeth, and the simpler infolding of the smaller maxillary teeth. C: interpretation of the cross-section in B. Light grey areas indicate dentine, while darker grey areas indicate alveolar bone. The thick black lines represent the axes of the folds. D: cross-section taken near the midpoint of the enlarged maxillary tooth and towards the tip of the smaller maxillary tooth showing a distinct lack of dentine infolding in both of the teeth. E: interpretation of the cross-section in D. Light grey areas indicate dentine, while the thick black lines represent the axes of the folds. de, dentine; m, maxilla; pc, pulp cavity.

Interestingly, the dentine in the enlarged maxillary teeth of *Colobomycter pholeter* shows a folding pattern that is quite distinct from that seen in the enlarged premaxillary tooth. Thin sections at the base of this tooth reveals that the dentine is highly folded, however, unlike in the enlarged premaxillary tooth it is much more irregular. Folds are still highly convoluted, but do not consist of lamellae, because the surrounding attachment tissues invade the centers of the folds (Figure 4B, C). In the midsection of the tooth, the irregular folding seen lower in the tooth is entirely absent and the dentine forms a circular outline of the tooth (Figure 4D, E).

The smaller teeth of *C. pholeter* do not show the complex infolding of the enlarged teeth, although they still clearly show the presence of infolding, albeit of a much simpler nature (Figure 4B, C). This suggests that, much like in temnospondyl anamniotes [34], the number and complexity of folds observed in the teeth of *C. pholeter* increases with tooth size.

**Figure 5:**
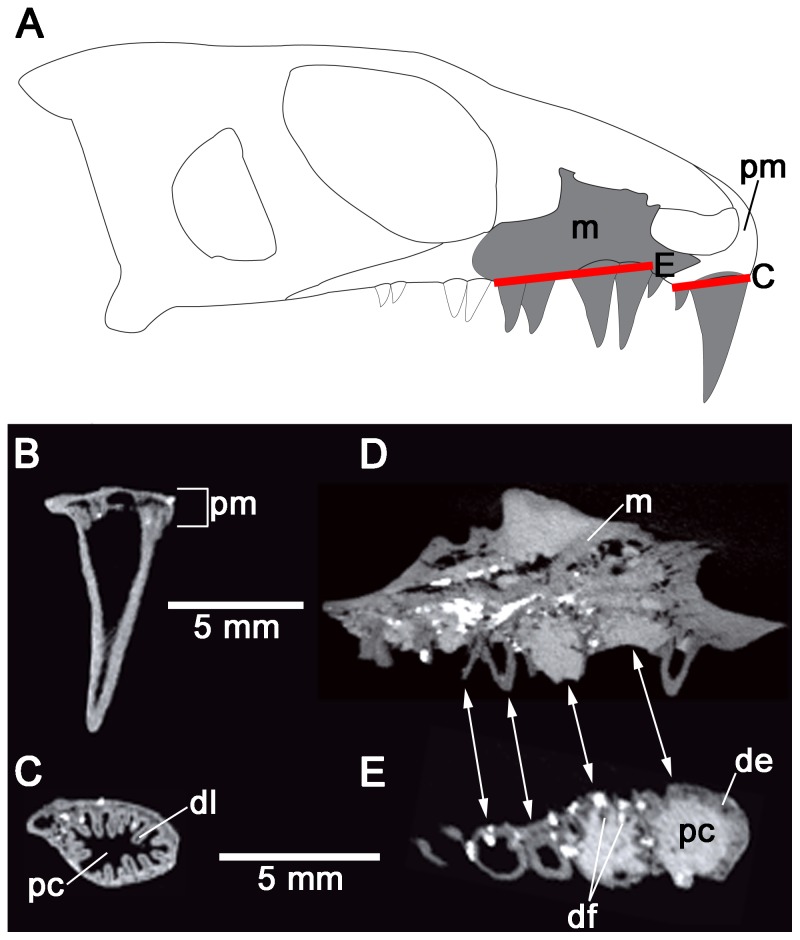
Cross-sectional and longitudinal views of the maxillary and premaxillary dentition of *Colobomycter pholeter* (OMNH 73535) obtained via X-ray computed tomography (CT) scans. A: diagram indicating from what parts of maxilla and premaxilla the virtual sections were obtained. B: virtual long-section of the enlarged premaxillary tooth showing its shallow implantation within the premaxilla. C: virtual cross-section of the base of the enlarged premaxillary tooth, which clearly shows the tight infolding of the dentine and the lamellae. D: virtual long-section of the maxillary dentition, shows their shallow implantation within the maxilla regardless of size. E: virtual cross-section of the bases of the maxillary dentition showing the complex dentine infolding of the enlarged maxillary teeth. The looser dentine infolding of the smaller maxillary teeth cannot be seen. de, dentine; dl, dentine lamellae; df, dentine fold; m, maxilla; pc, pulp cavity; pm, premaxilla.

CT scans of the teeth of another specimen of *Colobomycter pholeter* (OMNH 73535) revealed that it was possible to clearly view the infolding in the enlarged maxillary and premaxillary teeth (Figure 5). However, aside from being able to see the gross morphology of the folds, finer details such as the structural differences between the plicidentine in the enlarged premaxillary tooth and the enlarged maxillary teeth could not be observed. It was also difficult to see the folds of the smaller teeth, due to their small size and the limits on the resolution of the CT scans. The CT scans also indicated that both the enlarged and smaller teeth of *C. pholeter* are very shallowly implanted in a low, saucer-shaped alveolus.

Three other parareptiles, all from the same locality as *Colobomycter pholeter*, and considered to be fairly closely related to the latter, have yielded sufficient materials to make detailed study of their dentition possible. As is the case with *C. pholeter*, these are all small predators, and show various levels of heterodonty.

### 1) *Delorhynchus*

**Figure 6:**
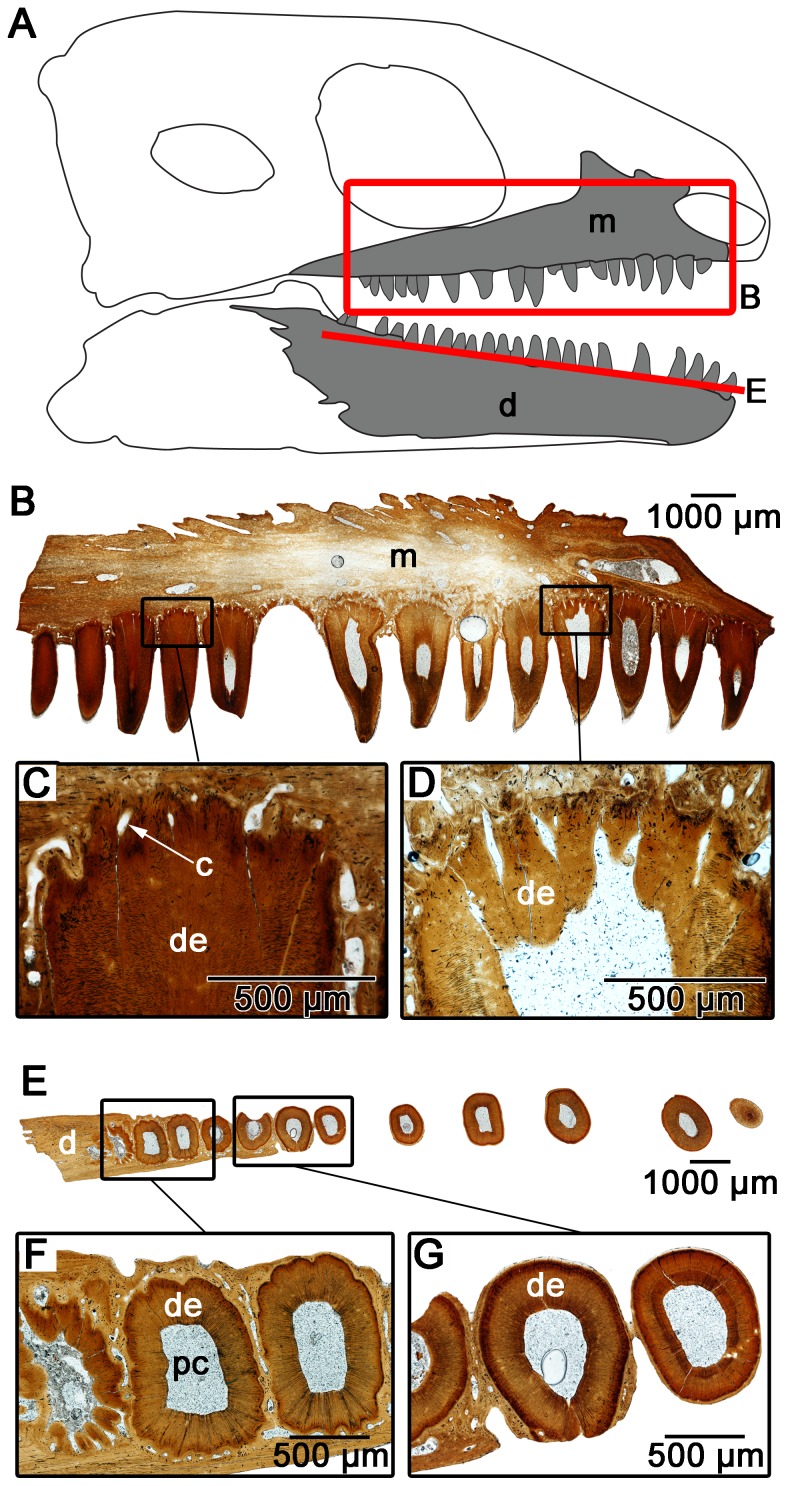
Cross-sectional and longitudinal views of the teeth of a new species of *Delorhynchus*. A: diagram indicating from what parts of the maxilla and dentary the histological sections were taken. B: long-section of the maxilla (ROM 67372). C: close up view of one of the posterior maxillary teeth from B. Shows the presence of radial canals surrounded by dentine at the base of the tooth. D: close up view of one of the anterior maxillary teeth from B. Shows how far the dentine folds project into the pulp cavity E: cross-section of the dentary (ROM 67373). F: close up view of some of the dentary teeth from E. Shows the loose dentine folding that is found at the base of the teeth. G: close up view of some of the dentary teeth from E. Shows that as you move towards the tip of the teeth the dentine loses its infolding. c, canal; d, dentary; de, dentine; m, maxilla; pc, pulp cavity.

The sister taxon of *Colobomycter pholeter*, *Delorhynchus* possesses a homodont dentition composed of simple conical teeth. The thin sections of the maxillary teeth in *Delorhynchus* reveal the presence of plicidentine (Figure 6), but not with the same degree of complexity seen in the enlarged teeth of *C. pholeter*. It is much more reminiscent of the plicidentine that was observed in smaller homodont teeth of *C. pholeter*. Longitudinal sections through a maxilla of *Delorhynchus* show that plicidentine is restricted to the bases of the teeth, well below the jaw line (Figure 6B–D). The longitudinal sections also show that the dentine folds encircle several canals, which extend from the exterior of the tooth base to the pulp cavity. These canals are very similar to those observed by Cabreira and Cisneros [12] in their longitudinal sections of the procolophonid *Soturnia caliodon*, and by Pretto et al. [30] in the longitudinal sections of the mesosaur *Stereosternum tumidum*. This suggests that the teeth of these parareptiles would also have exhibited loose folding of the dentine around these canals. In transverse sections, the dentine consists of loose folds and therefore does not possess lamellae, or any sort of branching. Deeper sections through individual teeth reveal the same systems of canals extending into the pulp cavity (Figure 6F). Overall, the plicidentine of the homodont dentition in *Delorhynchus* is similar to that seen in the small homodont teeth of *C. pholeter*.

### 2) *Microleter mckinzieorum*

**Figure 7:**
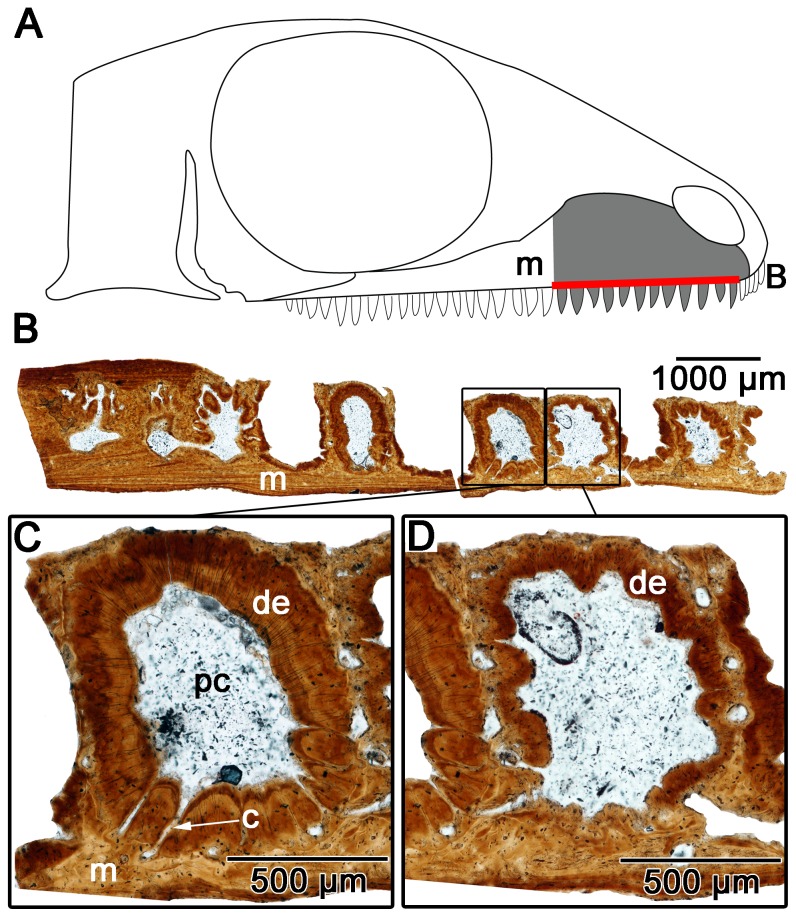
Cross-sectional views of the maxillary teeth of *Microleter mckinzieorum* (ROM 67375). A: diagram indicating from what parts of the maxilla the histological sections were taken. B: cross-section of the maxilla. C: close up view of one of the maxillary teeth from B. Shows the presence of radial canals in the dentine. D: close up view of one of the maxillary teeth from B. Shows the loose dentine infolding that is found at the base of the teeth. c, canal; de, dentine; m, maxilla; pc, pulp cavity.

Despite being outside of the clade Lanthanosuchoidea the thin sections of the simple homodont maxillary teeth of *Microleter mckinzieorum* show the presence of plicidentine reminiscent of that found in *Delorhynchus*, and the small homodont teeth of *Colobomycter pholeter* (Figure 7). The teeth in *M. mckinzieorum* also show the presence of a canal system similar to that observed in *Delorhynchus*. The midsection of the teeth reveals that loose folding is still present, although the canals are absent.

### 3) *Feeserpeton oklahomensis*

**Figure 8:**
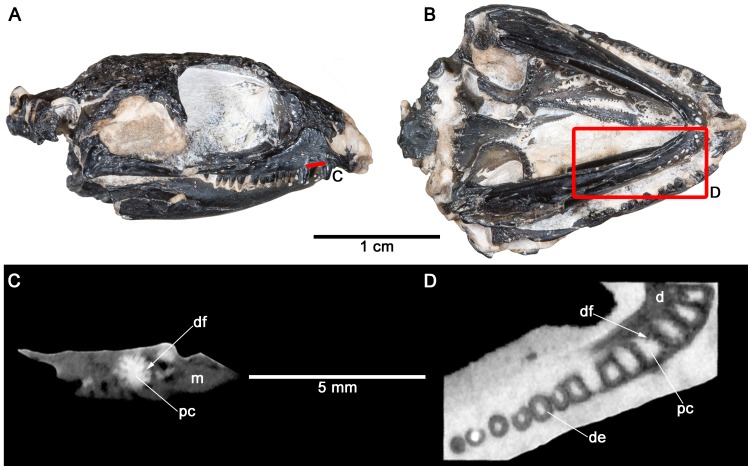
Cross-sectional views of the maxillary and dentary dentition of *Feeserpeton oklahomensis* (OMNH 73541) obtained via X-ray computed tomography (CT) scans. A: skull of *F. oklahomensis* in right lateral view showing from where the virtual sections of the maxilla were taken. B: skull of *F. oklahomensis* in ventral view showing from where the virtual sections of the dentary were taken. C: virtual cross-section of the base of one of the enlarged maxillary teeth showing the presence of tight dentine infolding. D: virtual cross-section of the bases of the dentary teeth showing the presence of dentine infolding. d, dentary; de, dentine; df, dentine fold; m, maxilla; pc, pulp cavity.

*Feeserpeton oklahomensis* exhibits a heterodont dentition consisting of simple conical teeth, three enlarged teeth on the maxilla, and two on the dentary (Figure 8A). *F. oklahomensis* was the only parareptile that was not examined histologically, because it is currently only known from the holotype (OMNH 73541). Since CT scans were shown to be useful in identifying plicidentine in *Colobomycter pholeter*, this technique was also applied to the holotype of *F. oklahomensis*. The CT scans revealed that the teeth of *F. oklahomensis* possess extensively folded dentine that is found at the bases of the enlarged maxillary and dentary teeth (Figure 8B, C), reminiscent of the folding observed in *Delorhynchus* and *Microleter mckinzieorum*. Due to the resolution of the CT scans it could not be determined if the folds of the enlarged teeth were lamellae as in the enlarged premaxillary tooth of *C. pholeter*. The midsection of the teeth reveals that the folding stops and unfolded dentine makes up the rest of the teeth. The CT scans did not have the resolution to confidently identify plicidentine within the smaller teeth.

### *Bolosaurus*

**Figure 9:**
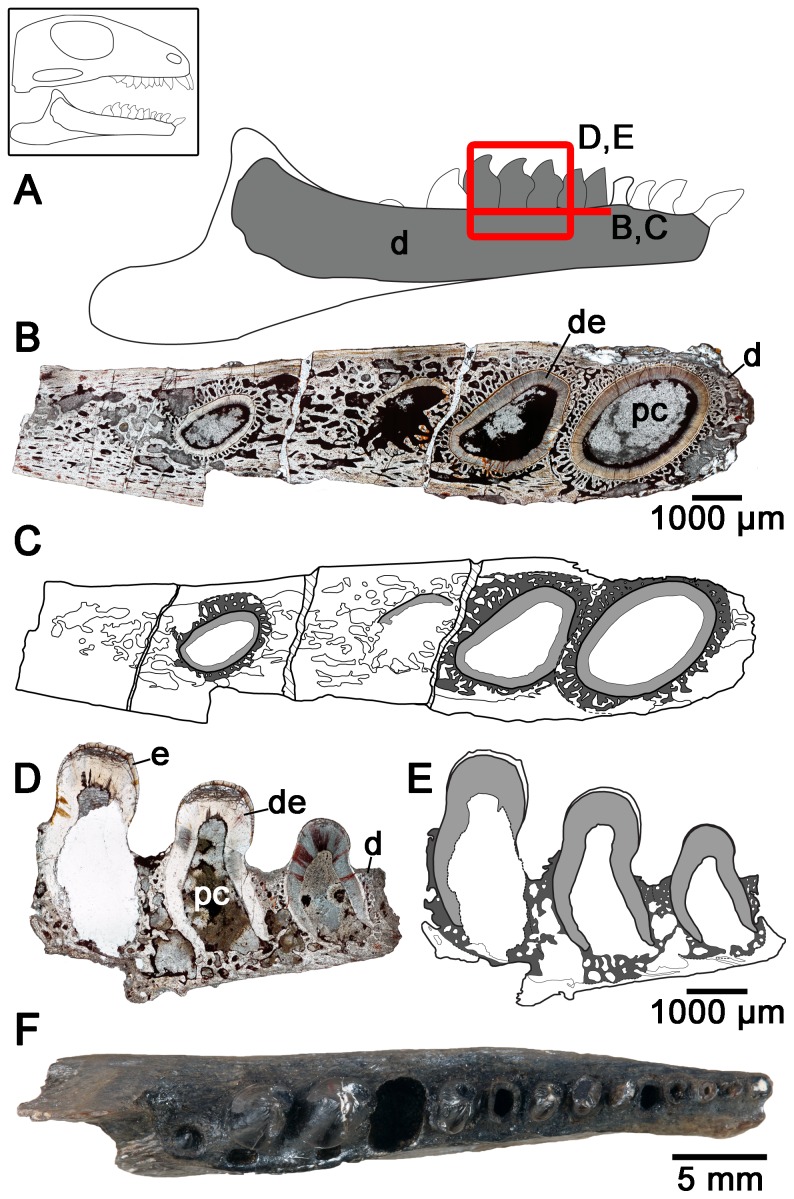
Cross-sectional and longitudinal views of the dentary teeth of *Bolosaurus*. A: diagram indicating from what parts of the dentary the histological sections were taken. B: cross-section of a *Bolosaurus striatus* dentary fragment (StlPB-R 636), showing a complete lack of any dentine infolding. C: interpretation of the cross-section in B. Light grey areas indicate dentine, while darker grey areas indicate alveolar bone. D: long-section of a *B. striatus* dentary fragment (StlPB-R 637), showing the deep implantation of the associated teeth. E: interpretation of the long-section in D. Light grey areas indicate dentine, while darker grey areas indicate alveolar bone. The white area on the crowns of some of the teeth indicates enamel. F: Occlusal view of the dentary of *Bolosaurus grandis* showing the deep sockets of the teeth. d, dentary; de, dentine; e, enamel; pc, pulp cavity.

Another parareptile, *Bolosaurus grandis* is present in the Dolese Brothers Limestone Quarry, near Richards Spur, but this material is too rare for sectioning and destructive analysis. However, this species of bolosaurid has the same kind of tooth attachment as another species of *Bolosaurus* commonly found in many localities in North America, *B. striatus* (Figure 9). The latter has slightly smaller dentition than *B. grandis*, and varies slightly in the pattern of the striations on the crown from those on *B. grandis*, but these differences are subtle, and are not expected to affect the presence or absence of plicidentine.

Bolosaurids are distinct from other parareptiles in having a highly specialized, heterodont dentition that was adapted to dental occlusion [33], [35]. *Bolosaurus striatus* exhibits the bulbous laterally expanded teeth common to all bolosaurids (Figure 9). External plication of the tooth base, often a telltale sign of plicidentine [15], [23], is not present on the teeth of *B. striatus*. Cross-sections taken below the alveolar margin of the dentary show a distinct lack of folding of the dentine, a stark contrast to what was observed in *Colobomycter pholeter*. The cross-sections of the tooth bases of *B. striatus* are oval-shaped, with their long axes oriented diagonal to the anteroposterior axis of the dentary (Figure 9B, C). The dentine tubules of each tooth base extend parallel to one another in a straight line from the external surface of the dentine wall to the pulp cavity, suggesting that there were no folds of the dentine as it developed in each tooth. Longitudinal sections through a dentary fragment of *B. striatus* show the distinct crowns of the teeth capped in thick layers of enamel. The size of the roots in comparison to the crowns shows that the teeth were deeply implanted into the jaw (Figure 9D, E). The teeth were held in place by extensive amounts of attachment tissue, presumably alveolar bone, sensu LeBlanc and Reisz [16]. The bases of the teeth are open, and show no evidence of complex infoldings towards the base.

## Discussion

### Identification of plicidentine in CT scans

CT scanning has the potential to reveal dental variation in specimens that are not amenable to destructive sampling, and is thus a potentially useful technique for examining plicidentine in vertebrates. Prior to this study, only Kearney and Rieppel [24] in their description of squamate plicidentine had used CT scans and compared them to histological samples. Here we present a comparison of CT scans of the dentition of *Colobomycter pholeter* with histological thin sections of its teeth in order to show the benefits and limitations of using CT scans for determining the fine structure of teeth, including the presence of plicidentine within teeth. *C. pholeter* is an ideal sample to evaluate the utility of this technique because of the fine-scale variation in the infolding patterns of its maxillary and premaxillary teeth. The thin sections of the teeth of *C. pholeter* clearly demonstrate the presence of complex plicidentine, and using CT scans with appropriate imaging software revealed the same folding pattern of the dentine (Figure 5). This technique was then applied to material that could not be histologically sampled, specifically the holotype and only known specimen of *Feeserpeton oklahomensis* (Figure 8).

The available evidence shows that this technique is useful for determining the gross morphology of the dentine within a tooth, but comparisons with histological thin sections indicate that the micro CT should be used with caution as it does have limitations. The CT scans of *C. pholeter* lacked the resolution required to identify plicidentine near the tip of the crown in the enlarged premaxillary tooth, whereas plicidentine was clearly visible in the histological thin sections of that area. Plicidentine within the smaller teeth was very difficult to see due to the size of the teeth and the resolution of the CT scans. This method does not make it possible to make out finer details of the dentine tubules, which highlight the folds in the histological thin sections. Despite these restrictions micro CT offers a method for determining whether or not plicidentine is present in a tooth, and can provide limited information about the complexity of the folds.

### Functional significance of plicidentine within Parareptilia

There have been several hypotheses proposed for the functional significance of plicidentine. One of the oldest hypotheses is that it strengthens the tooth base without increasing the amount of mineralized tissue used [36], [37]. Some argue that plicidentine also increases the surface area for attachment tissues [25], [36]. Scanlon and Lee [25] have also suggested that it allows for slight flexibility of the tooth base to absorb shock during feeding. It is worth noting that the functional purpose of plicidentine could be the result of interplay between these hypotheses, as they are not necessarily mutually exclusive [15].

Given the diversity of infoldings in this small sample of parareptiles, it is difficult to attribute a single function to the presence of plicidentine in Parareptilia. However, it appears that parareptiles with deeply implanted teeth, such as *Bolosaurus striatus*, lack plicidentine, whereas parareptiles with shallowly implanted teeth can exhibit very complex plicidentine, especially in larger teeth. Longitudinal thin sections of the teeth of *B. striatus* show that the teeth are deeply implanted within the tooth bearing elements, and transverse thin sections of its teeth show that they lack plicidentine. By comparison, CT scans of *C. pholeter* show that the teeth are very shallowly implanted, merely resting on the tooth-bearing surface, and thin sections of these teeth show very complex plicidentine, particularly at the base. This suggests that the complex, tightly folded plicidentine observed in the teeth of *C. pholeter* is probably associated with an increase in the surface area for attachment to compensate for their shallow implantation, as is observed in some teleostean fish [38], [39], whereas in *B. striatus* plicidentine is not necessary due to the deep implantation of the teeth. In order for enlarged maxillary or premaxillary teeth, such as those in *C. pholeter*, to be supported without folded dentine, the skull would require extensive modification. For example, saber teeth in mammals are very deeply implanted with the root extending very far into the skull; this deep implantation requires numerous modifications to the architecture of the skull in order to accommodate them [40].Thus it seems that an increase in surface area to accommodate the enlarged dentitions in *C. pholeter* is a reasonable hypothesis.

It is very important to note that *Colobomycter pholeter* exhibits different types of folding depending on the tooth position, suggesting that the various shapes and sizes of teeth served different functions, and may have had a significant impact on the degree of folding associated with the plicidentine. The enlarged premaxillary tooth, which possesses very tight folds for its entire length, probably served a different purpose than the paired large maxillary teeth that exhibit very convoluted plicidentine at their bases. However, there is currently little that can be said about the reason for this difference beyond the hypothesis that the enlarged teeth of *C. pholeter* played different roles than the small homodont teeth. Planned Finite Element Analyses of these teeth may help in resolving this interesting problem.

The most common type of plicidentine observed in our study was the loosely folded type seen in the new species of *Delorhynchus* and *Microleter mckinzieorum*. The loose folds are only found at the base of the teeth and are punctuated by radial canals. These canals are similar to what is observed in the teeth of the reptile *Captorhinus aguti* [26], their function is presumably to allow the entry of blood vessels into the pulp cavity [15]. The functions of the loose folds cannot be determined at this time, but may have had a role similar to that of the tight folds found in the enlarged teeth of *Colobomycter pholeter*, except on a smaller scale.

### Phylogenetic utility of plicidentine within Parareptilia

Many previous phylogenetic analyses of Parareptilia have used a character that concerns the presence/absence of labyrinthodont infolding (i.e. plicidentine) in teeth [1], [2], [19]. This character has generally been coded as present for many of the anamniote outgroup taxa, and has been coded as absent for amniote taxa [1], [2], [19]. This character was initially coded in this manner largely due to the previous lack of knowledge regarding dentine folding within amniotes. Thus, many of the codings of older analyses were carried over to subsequent studies without reevaluation. The apparent lack of plicidentine in most anatomical studies of parareptiles is most likely the result of the fact that most of the folded portions of the tooth roots in parareptiles were found below the jawline in our analysis, with no obvious external plications being visible on the teeth. The only exception to this was the extensive grooves along the enlarged teeth of *Colobomycter pholeter* (Figure 5). However, as there is now evidence supporting the presence of plicidentine within many other amniote taxa [14], [15], [21]–[26], this particular character warrants reevaluation within Parareptilia in determining its phylogenetic utility.

Our study, combined with the results of others [12], [30], indicates that there is a wide spectrum of dentine folding across parareptilian taxa (Figure 1), making this character much more complex than a simple binary character: presence or absence of plicidentine. Perhaps most important is the discovery that plicidentine can vary significantly in adjacent teeth in the same taxon, as exemplified by *Colobomycter pholeter*. Related to the plicidentine variation in *C. pholeter* and the other parareptiles is that larger teeth exhibit more complex dentine folding patterns (as in labyrinthodont anamniotes), which suggests that different types of plicidentine are more related to tooth size rather than phylogeny. Thus, we recommend that for the time being, characters involving plicidentine should be removed from phylogenetic analyses of Parareptilia, at least until there is more complete data on the distribution of the feature within the clade. The variability of plicidentine within the teeth of a single taxon must also be examined prior to any future inclusion of plicidentine characters into parareptilian phylogenetic analyses.

## Conclusions

Comparisons of several coeval parareptilian taxa reveal that plicidentine is much more common within Parareptilia than previously thought. Different members of the clade exhibits a wide array of dentine folding, ranging from a lack of folding entirely, as seen in *Bolosaurus*, to very complex folding that is reminiscent of the plicidentine observed in temnospondyl amphibians. We also describe for the first time variation in plicidentine within a single taxon; *Colobomycter pholeter*, which exhibits three different folding patterns depending on the tooth position and size. This variation also reinforces the likelihood that heterodonty in *C. pholeter* is associated with different functions for the different teeth. The presence of plicidentine in parareptiles also supports the hypothesis that one function of plicidentine is to increase the surface area for tissue attachment between dentine and the periodontium. However, this function can only be attributed to those teeth that are shallowly implanted and exhibit the most extensive infolding patterns towards the base of the tooth, where it interacts with the attachment tissues. Bolosaurids have deeply implanted teeth that do not exhibit plicidentine. It is likely that the relationship between the dentine and periodontium in bolosaurids may be related to the evolution of dental occlusion [35]. With the information gained from this study it is now apparent that the presence of plicidentine in parareptiles has a complicated evolutionary history. However, the large variability of plicidentine within the clade, its variability within a single taxon, and its apparent relation to tooth size diminishes the phylogenetic utility of plicidentine. Given the problematic nature of this feature of parareptilian dentitions, we suggest that it should not be used as a character within phylogenetic analyses of Parareptilia, at least until we gain a better understanding of the distribution and evolutionary history of this feature.
